# Complete Genome Sequences of Hydrogenotrophic Denitrifiers

**DOI:** 10.1128/mra.01020-21

**Published:** 2022-01-13

**Authors:** Clara Duffner, Susanne Kublik, Bärbel Fösel, Michael Schloter, Stefanie Schulz

**Affiliations:** a Chair of Soil Science, TUM School of Life Sciences Weihenstephan, Technical University of Munich, Freising, Germany; b Research Unit Comparative Microbiome Analysis, Helmholtz Zentrum München Deutsches Forschungszentrum für Gesundheit und Umwelt, Neuherberg, Germany; University of Delaware

## Abstract

Hydrogenotrophic denitrifiers are important bacteria for nitrate removal in wastewater and aquifers. Here, we report the complete genome sequences of three hydrogenotrophic denitrifiers, namely, Dechloromonas denitrificans strain D110, Ferribacterium limneticum strain F76, and Hydrogenophaga taeniospiralis strain H3, all of which were isolated from a nitrate-polluted aquifer in Bavaria (Germany).

## ANNOUNCEMENT

We isolated three hydrogenotrophic denitrifiers after enrichment from sediment (H3) and groundwater (D110 and F76) samples from a highly nitrate-polluted aquifer in the Hohenthann region of southeast Germany (1). Samples were obtained as described (2). Enrichment and isolation were performed under anoxic conditions with a 60% H_2_/10% CO_2_/30% N_2_ atmosphere. Cultivation was done on mineral medium agar plates after all available nitrate and nitrite had been reduced. The mineral medium contained basal medium (3), 30 mM NaHCO_3_ buffer, 1.5 mM NaNO_3_, 0.2% trace element solution (4), 0.1% selenite tungsten solution, and 0.1% vitamin solution (5). The isolates obtained were phylogenetically assigned by Sanger sequencing of the full 16S rRNA gene (primer pair 27f/1492r [6]) and aligned against the NCBI rRNA and internal transcribed spacer (ITS) databases with the nucleotide Basic Local Alignment Search Tool (BLASTn) (v2.10.0).

The complete genome sequences of Dechloromonas denitrificans D110, Ferribacterium limneticum F76, and Hydrogenophaga taeniospiralis H3 were obtained using single-molecule real-time (SMRT) cells and the Sequel system (Pacific Biosciences [PacBio], CA, USA). The DNA was extracted from 4.5 × 10^9^ cells that had been grown in R2A medium at 30°C using the anion-exchange-based Genomic-tip (20/G) kit (Qiagen, Hilden, Germany). The multiplexed microbial libraries were prepared according to the procedure and checklist of the SMRTbell Express template preparation kit v2.0 (product number 101-696-100, v6 [March 2020]; PacBio). The genomic DNA was sheared to 9.5- to 12.6-kb-long fragments using g-TUBEs (Covaris, MA, USA) and further processed without additional size selection. The libraries, with a maximum expected genome size of 33 Mb, were loaded onto two SMRT cells at concentrations of 3 pM and 6 pM. Libraries were immobilized on the SMRT cells (2 h), preextended (2 h), and then sequenced on the Sequel system using v3.0 chemistry with a movie time of 10 h. The data were demultiplexed and the genomes were assembled using the HGAP4 pipeline embedded in SMRT Link v8.0.0.80529 (PacBio), with a seed coverage of 30×.

Details on the aligned subreads are summarized in Table 1. The genomes of D. denitrificans D110 and F. limneticum F76 comprised only one large contig each, which were circularized successfully with Circlator software v1.5.5 (7). The large contig of the H. taeniospiralis H3 genome was also circularized, while two additional small contigs of 5,700 and 2,750 bp could not be circularized.

**TABLE 1:** SMRT Link-polished assembly results for the sequenced isolates, as well as the number of CDSs, 16S rRNAs, and tRNAs determined with NCBI PGAP

Isolate	No. of realigned subreads	Mean subread length (bp)	Mean coverage (fold)	No. of contigs	Maximum contig length (bp)	No. of CDSs	No. of 16S rRNAs	No. of tRNAs
D. denitrificans D110	525,543	4,805	511	1	4,619,273	4,301	4	63
F. limneticum F76	266,526	4,629	259	1	4,419,139	4,128	3	58
H. taeniospiralis H3	233,679	5,387	206	3	5,275,671, 5,700, and 2,750	4,935	3	50

According to CheckM software v1.1.2 (8), all three genome sequences were at least 99.2% complete, with a maximum contamination of 0.93% in the genome of H. taeniospiralis H3. The GC contents were similar for all three genomes, i.e., 62% for D. denitrificans D110, 60% for F. limneticum F76, and 67% for H. taeniospiralis H3. The genomes were phylogenetically assigned with the Type Strain Genome Server (TYGS) from DSMZ (9) and functionally annotated by the NCBI Prokaryotic Genome Annotation Pipeline (PGAP) (https://www.ncbi.nlm.nih.gov/genome/annotation_prok). The latter predicted 4,301 coding sequences (CDSs) in the genome of D. denitrificans D110, 4,128 CDSs in F. limneticum F76, and 4,935 CDSs in H. taeniospiralis H3 (Table 1). All genomes included genes coding for denitrification reductases, hydrogenases, and ribulose-1,5-bisphosphate carboxylase-oxygenase (RubisCO), which are all required for hydrogenotrophic denitrification.

### Data availability.

The assembly and annotation, as well as the raw reads, for the three genomes are available via BioProject PRJNA727717. The NCBI assembly accession numbers for the genomes are GCA_020510585.1 for F. limneticum F76, GCA_020510685.1 for D. denitrificans D110, and GCA_020510445.1 for H. taeniospiralis H3. The respective raw reads can be found under the accession numbers SRR16235514, SRR16235513, and SRR16235512.
